# Emotion regulation and psychological wellbeing in university students: anxiety, depression, stress, and life satisfaction

**DOI:** 10.3389/fpsyg.2026.1913567

**Published:** 2026-08-31

**Authors:** José María Augusto-Landa, Claudia De Barros-Camargo, Antonio Hernández-Fernández, David Molero, Rubén Juárez-Cádiz

**Affiliations:** 1 Department of Psychology, Area of Social Psychology, University of Jaén, Jaén, Spain; 2 Department of Research Methods and Diagnosis in Education I, National Distance Education University (UNED), Madrid, Spain; 3 Department of Pedagogy, University of Jaén, Jaén, Spain; 4 Department of Education, Research Methods and Educational Diagnosis, Lifelong Educational Research: Neuropedagogical Integration, University of Jaén, Jaén, Spain; 5 Department of Engineering, San Pablo CEU University, Madrid, Spain

**Keywords:** anxiety, cognitive reappraisal, depression, emotion regulation, expressive suppression, life satisfaction, stress, university students

## Abstract

Emotion regulation is relevant to psychological adjustment among university students, particularly in contexts characterized by academic pressure and increasing demands for autonomy. This cross-sectional study examined associations between cognitive reappraisal, expressive suppression, depression, anxiety, stress, and life satisfaction in 106 undergraduate students enrolled in a Social Education degree at a public university in Andalusia, Spain. Participants completed the Emotion Regulation Questionnaire, the Satisfaction With Life Scale, and the Depression Anxiety Stress Scales-21. Internal consistency was high across the six study dimensions (Cronbach's α =.846–.921; McDonald's ω =.848–.921). Life satisfaction was negatively correlated with depression, anxiety, and stress, and the three distress dimensions were positively interrelated. In gender-adjusted hierarchical regressions, emotion-regulation scores explained additional variance in anxiety and stress, but not in depression or life satisfaction. Several associations were opposite to the directions commonly reported in the literature: cognitive reappraisal was positively associated with anxiety, whereas expressive suppression was negatively associated with anxiety and stress. The item-level structural model produced mixed fit evidence: RMSEA, CFI, and TLI/NNFI were favorable, but SRMR, GFI, AGFI, NFI, and RFI indicated important limitations. Because indirect effects were not estimated and the data were cross-sectional, the model does not demonstrate mediation or causal pathways. The findings should therefore be regarded as sample-specific associations requiring replication and a complete scoring audit.

## 1 Introduction

The transition to university constitutes a critical developmental period in which academic, social, emotional, and personal demands converge. Entering higher education usually involves substantial changes in study habits, learning routines, interpersonal relationships, academic expectations, autonomy, time management, and future-oriented decision-making. Although this transition may promote intellectual growth, personal maturity, and professional identity development, it may also increase students' exposure to emotionally demanding situations. When these demands exceed perceived personal and contextual resources, they may be expressed through stress, anxiety, emotional exhaustion, concentration difficulties, irritability, reduced perceived competence, sleep problems, and lower life satisfaction (Restrepo et al., 2020; Khorasani et al., 2023; Samaha and Hawi, 2016). Consequently, university wellbeing should not be conceptualized merely as the absence of psychopathological symptoms, but rather as a dynamic balance between academic demands, personal coping resources, social support, perceived self-efficacy, emotional competence, and the capacity to adapt to changing educational environments (Conley and French, 2014; Eisenberg et al., 2009).

Recent multicentre evidence reinforces the importance of academic pressure and coping resources in this population. In a sample of 2,103 Italian university students, Micheluzzi et al. (2025) identified moderate-to-high perceived stress, with academic demands among the most prominent stressors and small but relevant differences according to sex, age, year of study, and discipline. Their findings support interpreting student stress within a broader academic and institutional context rather than solely as an individual deficit.

Depression, anxiety, and stress are among the most relevant indicators for examining psychological adjustment in university populations. Although these constructs are theoretically distinguishable, they frequently co-occur and share transdiagnostic mechanisms related to negative emotionality, heightened affective reactivity, persistent worry, avoidance, rumination, and difficulties in recovering emotional balance after adverse or demanding experiences (Bullis et al., 2019; Liu et al., 2022). In university students, these forms of psychological distress may interfere with academic performance, motivation, social participation, decision-making, perceived quality of life, and persistence in higher education (Eisenberg et al., 2009). Previous research has consistently shown that university students are particularly vulnerable to anxiety, depressive symptoms, and stress, especially when they face academic overload, examinations, uncertainty about the future, social comparison, or difficulties in managing time and responsibilities (Asif et al., 2020; Shaffique et al., 2020). Therefore, identifying modifiable psychological mechanisms that contribute to psychological adjustment is essential for designing preventive interventions and institutional support programs in higher education.

One of the most relevant mechanisms in this regard is emotion regulation. According to the process model proposed by Gross (2002), emotion regulation refers to the processes through which individuals influence which emotions they have, when they have them, and how these emotions are experienced and expressed. This theoretical framework distinguishes between strategies focused on the antecedents of the emotional response and strategies focused on the response itself. Cognitive reappraisal is an antecedent-focused strategy that involves modifying the interpretation or meaning attributed to a situation in order to change its emotional impact. For example, a student may interpret an examination not only as a threatening event, but also as an opportunity to demonstrate learning or identify areas for improvement. Expressive suppression, by contrast, is a response-focused strategy that consists of inhibiting or modulating the external expression of emotion once the emotional response has already been activated (Gross and John, 2003).

The distinction between cognitive reappraisal and expressive suppression has been central to the study of emotional functioning and psychological wellbeing. Cognitive reappraisal has generally been considered a more adaptive strategy because it acts earlier in the emotion-generative process and may reduce emotional intensity before the response is fully consolidated. Expressive suppression, however, has often been described as a less adaptive strategy because it does not necessarily reduce the internal emotional experience and may require sustained cognitive and physiological effort (Gross, 2002; Gross and John, 2003). From this perspective, students who rely more frequently on reappraisal may be better able to reinterpret academic setbacks, interpersonal conflicts, or stressful situations in a way that reduces emotional burden. Conversely, students who frequently suppress emotional expression may appear externally controlled while maintaining high internal levels of distress.

Empirical evidence has generally supported this distinction. The meta-analysis conducted by Aldao et al. (2010) showed that emotion regulation strategies are differentially associated with psychopathological symptoms. Strategies such as cognitive reappraisal, acceptance, and problem solving tend to be negatively associated with psychological distress, whereas strategies such as rumination, avoidance, worry, catastrophizing, and suppression tend to be positively associated with anxiety, depression, and stress. Similarly, Hu et al. (2014) found that cognitive reappraisal is usually associated with more favorable mental health indicators, including higher wellbeing and life satisfaction, whereas expressive suppression may be associated with anxiety, negative affect, interpersonal difficulties, and lower psychological wellbeing. In the university context, these relationships are especially relevant because students are required to regulate emotions in situations involving academic evaluation, uncertainty, performance pressure, social adaptation, and perceived expectations from teachers, peers, and families.

Nevertheless, contemporary approaches have increasingly emphasized that emotion regulation strategies should not be interpreted in an overly rigid or dichotomous way. The adaptive or maladaptive value of a given strategy may depend on the context, the intensity of the emotional experience, the specific regulatory goal, the time at which the strategy is implemented, the frequency of use, and the flexibility with which the individual selects and changes strategies according to situational demands (Bonanno and Burton, 2013; Kelley et al., 2019). In this sense, a strategy such as expressive suppression may be functional in the short term when a student needs to maintain composure during an oral presentation, a clinical practice, or an examination. However, the chronic and inflexible use of suppression may become problematic if it prevents emotional processing, reduces authenticity in interpersonal relationships, or increases internal tension. Likewise, cognitive reappraisal is not always automatically protective, because it may coexist with high anxiety when it is used as an effortful attempt to manage situations already perceived as highly demanding or uncontrollable. This perspective of regulatory flexibility is particularly important in university students. The university environment requires students not only to regulate emotions, but also to select appropriate strategies according to the type of demand they are facing. A student experiencing academic overload may need to combine reappraisal with planning, problem solving, social support, and rest. A student facing interpersonal conflict may require emotional expression, perspective taking, and communication skills rather than mere suppression. Therefore, rather than classifying cognitive reappraisal and expressive suppression as universally adaptive or maladaptive, it is necessary to analyze how these strategies operate within each student's broader psychological and academic ecosystem. This approach allows a more nuanced understanding of the relationship between emotion regulation, psychological distress, and subjective wellbeing.

Life satisfaction is another key construct for understanding university wellbeing. It represents the cognitive component of subjective wellbeing and refers to the global evaluation that individuals make of their own lives according to their personal criteria Diener et al., (1985). In university students, life satisfaction is associated with academic achievement, perceived competence, future expectations, social support, emotional stability, autonomy, and coping. Several studies have shown lower life satisfaction among students reporting greater depression, anxiety, and stress (Samaha and Hawi, 2016; Liu et al., 2022). These relationships provide a rationale for evaluating emotion regulation and distress jointly, without assuming causal direction.

The theoretical relationship among emotion regulation, psychological distress, and life satisfaction suggests the value of examining both isolated bivariate associations and an integrated covariance structure. Depression, anxiety, and stress may be statistically intermediate between emotion-regulation scores and life satisfaction, but demonstrating such an indirect association requires a formal indirect-effect test and does not by itself establish temporal mediation. Personal, academic, and contextual variables such as resilience, social support, academic self-efficacy, perceived control, and coping style may also be relevant (Cohen and Wills, 1985; Connor and Davidson, 2003).

**Figure 1:**
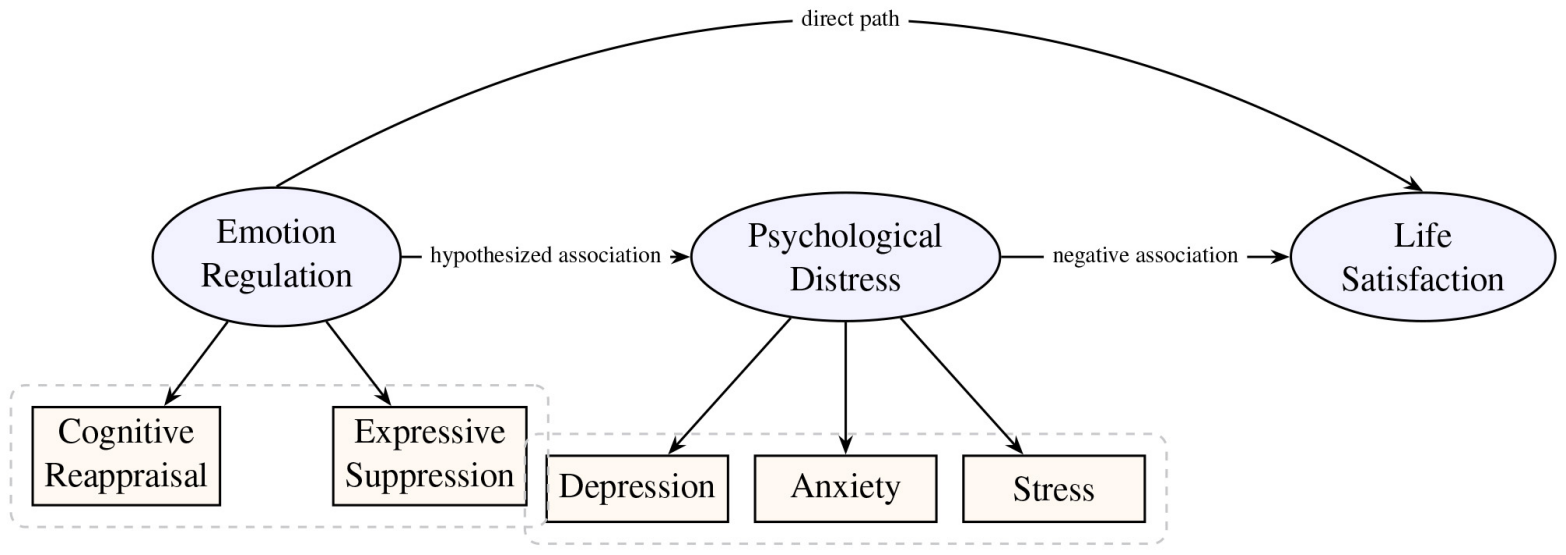
Hypothesized theoretical model of the relationships among emotion regulation, psychological distress, and life satisfaction.

Based on this theoretical and empirical background, the present study analyzes associations among cognitive reappraisal, expressive suppression, depression, anxiety, stress, and life satisfaction. It also examines the variance explained by emotion-regulation strategies in cross-sectional regression models and evaluates the fit of an integrated structural model. Figure 1 presents the hypothesized theoretical model.

The objectives were to analyze the relationships among emotion regulation, psychological distress, and life satisfaction; to examine whether cognitive reappraisal and expressive suppression explained variance in the four outcomes after adjustment for gender; and to evaluate the fit of the integrated structural model.

In accordance with the process model of emotion regulation and previous evidence, four hypotheses were formulated. H1: emotion-regulation strategies will be associated with anxiety, depression, stress, and life satisfaction. H2: greater cognitive reappraisal will be associated with higher life satisfaction. H3: cognitive reappraisal will be negatively, and expressive suppression positively, associated with depression, anxiety, and stress. H4: depression, anxiety, and stress will be negatively associated with life satisfaction. An indirect-association hypothesis is not retained because the archived analysis did not include formally estimated indirect effects and bootstrap confidence intervals.

For correspondence between the conceptual model and the analyses, H1–H4 were evaluated through correlations, gender-adjusted hierarchical regressions, and global structural-model fit. The arrows in Figure 1 represent hypothesized cross-sectional relations; they do not imply causal direction or demonstrated mediation.

## 2 Materials and methods

### 2.1 Design

A quantitative, non-experimental, cross-sectional, correlational design was used. No variables were manipulated and participants were not assigned to experimental conditions. The analyses examined associations among emotion regulation, psychological distress, and life satisfaction in a naturally occurring university context. Regression coefficients quantify conditional cross-sectional associations and explained variance; they are not interpreted as evidence of temporal prediction or causal effects.

All variables were measured at a single time point. Hierarchical regressions examined whether emotion-regulation scores accounted for additional variance in depression, anxiety, stress, and life satisfaction beyond gender. A structural equation model was also estimated to examine the statistical fit of the integrated theoretical model. Neither analysis establishes temporal ordering.

Given the absence of experimental manipulation, random assignment, and temporal sequencing, the findings are interpreted only as statistical associations. They do not establish directionality or causality.

Table 1 summarizes the main methodological characteristics of the study design.

**Table 1:** Summary of the methodological design.

Design component	Description
General approach	Quantitative, non-experimental, cross-sectional, and correlational study.
Research context	Natural university setting, without manipulation of variables or experimental assignment.
Temporal structure	Cross-sectional data collection at a single time point during the 2025–2026 academic year.
Predictor variables	Emotion regulation strategies: cognitive reappraisal and expressive suppression.
Criterion variables	Depression, anxiety, stress, and life satisfaction.
Control variable	Gender was included as a covariate in the hierarchical regression models.
Main analyses	Descriptive statistics, Pearson correlations, hierarchical regressions, and structural equation modeling (SEM).
Inferential scope	Results support associative interpretations only; temporal and causal conclusions are not warranted.

### 2.2 Participants

The sample consisted of 106 undergraduate university students enrolled in a Social Education degree at a public university located in southeastern Andalusia, Spain. Participants were recruited through non-probabilistic convenience sampling based on accessibility and voluntary participation. No a priori power analysis was documented. Accordingly, the sample should be regarded as modest, especially for an item-level SEM, and the precision and stability of the structural estimates may be limited.

All participants were students from the same degree program, which provided a relatively homogeneous academic context for the analysis. This homogeneity reduced variability associated with different fields of study, curricular structures, or institutional conditions. However, it also limits the generalizability of the findings to other degrees, universities, or student populations. Therefore, the results should be interpreted as evidence derived from a specific university sample rather than as estimates representative of the broader university population.

Regarding gender distribution, 85 participants identified as women (80.2%), 20 as men (18.9%), and one participant selected another option (0.9%). The mean age was 20.30 years (SD = 2.32). Gender was included as an exploratory covariate in the hierarchical regression models. Because the third category contained only one participant, it could not be estimated as a separate inferential group; the archived output does not contain sufficient information to reconstruct the exact contrast coding or its analytical treatment. Gender-adjusted coefficients should therefore be interpreted cautiously. The pronounced imbalance also limits generalizability.

Participation was voluntary and anonymous. Students were informed about the general purpose of the study, the confidential treatment of their responses, and their right to participate without any academic consequence. Only aggregated data were used in the statistical analyses. Table 2 summarizes the main demographic characteristics of the sample.

**Table 2:** Demographic characteristics of the sample.

Characteristic	Description
Sample size	106 undergraduate university students.
Academic program	Social Education degree.
Institutional context	Public university located in southeastern Andalusia, Spain.
Sampling procedure	Non-probabilistic convenience sampling based on accessibility and voluntary participation.
Gender distribution	85 women (80.2%), 20 men (18.9%), and 1 participant selecting another option (0.9%).
Age	*M* = 20.30 years; *SD* = 2.32.
Analytical consideration	Gender was included as a covariate in the hierarchical regression models.
Scope of generalization	Findings should be interpreted within the context of this specific university sample.

### 2.3 Instruments

Three standardized self-report instruments were used to assess emotion regulation, life satisfaction, and psychological distress. The selection of these instruments was based on their theoretical relevance to the proposed model, their frequent use in psychological and educational research, and the availability of Spanish adaptations with adequate psychometric evidence. Together, these measures allowed the study to examine both adaptive and maladaptive emotional processes, as well as positive and negative indicators of psychological adjustment in university students.

#### 2.3.1 Emotion Regulation Questionnaire (ERQ)

Emotion regulation was assessed using the Emotion Regulation Questionnaire developed by Gross and John (2003), in its Spanish adaptation by Cabello et al. (2013). Its 10 items are rated from 1, completely disagree, to 7, completely agree. Cognitive reappraisal was scored from items 1, 3, 5, 7, 8, and 10, and expressive suppression from items 2, 4, 6, and 9; no ERQ item was reverse-coded. Higher scores indicated greater use of the corresponding strategy. The original study reported Cronbach's alpha values of 0.79 and 0.73, respectively, and the Spanish adaptation provided evidence of the expected two-factor structure, reliability, and convergent validity (Gross and John, 2003; Cabello et al., 2013). In the present sample, internal consistency was high for cognitive reappraisal (α = 0.879, ω = 0.881) and expressive suppression (α = 0.859, ω = 0.861).

#### 2.3.2 Satisfaction With Life Scale (SWLS)

Life satisfaction was assessed with the five-item SWLS (Diener et al., 1985). All items are rated on a seven-point scale and contribute in the same direction; no item is reverse-coded. The mean item score was used, with higher values indicating greater life satisfaction. The original study supported a unidimensional structure and adequate internal consistency, and Spanish-language research has provided additional validity and reliability evidence (Diener et al., 1985; Vázquez et al., 2013). Present-sample internal consistency was high (α = 0.846, ω = 0.848).

#### 2.3.3 Depression Anxiety Stress Scales-21 (DASS-21)

Psychological distress was assessed using the DASS-21 (Antony et al., 1998), in its Spanish adaptation (Daza et al., 2002). Responses range from 0, did not apply to me at all, to 3, applied to me very much or most of the time. Depression comprised items 3, 5, 10, 13, 16, 17, and 21; anxiety items 2, 4, 7, 9, 15, 19, and 20; and stress items 1, 6, 8, 11, 12, 14, and 18. No DASS-21 item was reverse-coded. Mean item scores were used so that the reported values remained on the 0–3 response metric. The three-factor structure, convergent validity, and internal consistency have been supported in prior validation studies, including Spanish-speaking and Spanish university samples (Antony et al., 1998; Daza et al., 2002). Present-sample internal consistency was high for depression (α = 0.875, ω = 0.875), anxiety (α = 0.909, ω = 0.910), and stress (α = 0.921, ω = 0.921).

Table 3 summarizes the scoring and present-sample internal-consistency coefficients.

**Table 3:** Scoring and internal consistency of the study measures.

Dimension	Items	Response range	Cronbach's α	McDonald's ω
Cognitive reappraisal (ERQ)	6	1–7	0.879	0.881
Expressive suppression (ERQ)	4	1–7	0.859	0.861
Life satisfaction (SWLS)	5	1–7	0.846	0.848
Depression (DASS-21)	7	0–3	0.875	0.875
Anxiety (DASS-21)	7	0–3	0.909	0.910
Stress (DASS-21)	7	0–3	0.921	0.921

Coefficients were calculated for the present sample and are rounded to three decimal places.

The three instruments were treated as complementary measures within the proposed theoretical model. The ERQ provided information about the regulatory strategies used by students, the DASS-21 captured negative emotional functioning through depression, anxiety, and stress, and the SWLS assessed the positive cognitive evaluation of life. This combination made it possible to analyze whether emotion regulation strategies were associated with both psychological distress and subjective wellbeing, and whether distress-related variables could help explain the relationship between emotion regulation and life satisfaction.

### 2.4 Procedure

Data were collected during the 2025–2026 academic year, specifically in May 2026, using self-report questionnaires administered in a face-to-face academic context with online response. This mixed administration format made it possible to ensure that all participants received the same general instructions while allowing them to complete the instruments individually and digitally. The procedure was designed to minimize disruption to normal academic activity and to facilitate standardized data collection.

Before completing the questionnaires, students were informed about the general purpose of the study, the voluntary nature of their participation, the anonymous processing of their responses, and the confidential treatment of the information provided. They were also informed that participation or non-participation would not have any academic consequences. All participants provided informed consent before accessing the questionnaire battery. The protocol was approved by the Research Ethics Committee of the University of Jaén (approval code JUL.22/4-LÍNEA).

The questionnaire battery included the Emotion Regulation Questionnaire, the Satisfaction With Life Scale, and the Depression Anxiety Stress Scales-21. The approximate completion time was 15 minutes. Participants answered the items individually, without discussion with peers, and no identifying personal information was associated with the responses used for statistical analysis. Once data collection was completed, responses were coded, reviewed for consistency, and analyzed in aggregated form.

The data management process followed principles of anonymity, minimization, and confidentiality. Individual responses were not reported at any point, and the analyses were conducted only at group level. The dataset was used exclusively for the purposes of the present study. Table 4 summarizes the main stages of the research procedure.

**Table 4:** Main stages of the study procedure.

Stage	Description
Study presentation	Students were informed about the general aims of the study and the type of participation required.
Ethical approval	Research Ethics Committee of the University of Jaén; approval code JUL.22/4-LÍNEA.
Informed consent	Only students who provided informed consent completed the questionnaire battery.
Questionnaire administration	The instruments were administered in a face-to-face academic setting with online response.
Completion time	The approximate time required to complete the questionnaires was 15 minutes.
Data coding	Responses were coded without linking identifying personal information to the analytical dataset.
Data analysis	Data were analyzed in aggregated form using descriptive, correlational, hierarchical-regression, and structural analyses.

### 2.5 Statistical analysis

Statistical analyses were conducted using SPSS v26 and LISREL v8.80. Scale scores were computed using the item keys described above; none of the ERQ, SWLS, or DASS-21 items included in these keys required reverse coding. Cronbach's alpha and McDonald's omega were calculated for every study dimension. The retained output does not document item-level missingness, imputation, or complete-case decisions, and no unsupported missing-data procedure has been added retrospectively. This limitation is reported explicitly because it affects reproducibility.

First, descriptive statistics were calculated for the main study variables. Means and standard deviations were used to describe life satisfaction, depression, anxiety, stress, cognitive reappraisal, and expressive suppression. These analyses provided an initial profile of the emotional and psychological functioning of the sample and made it possible to identify which dimensions of psychological distress were more prominent among the participating students.

Second, Pearson correlation analyses were performed to examine the direction and magnitude of the bivariate associations among the variables. These analyses were used to test the expected relationships between life satisfaction and psychological distress, the interrelations among depression, anxiety, and stress, and the associations between emotion regulation strategies and the remaining psychological variables. Correlation coefficients were interpreted according to their magnitude, direction, and statistical significance.

Third, four hierarchical regression models were estimated, one for each outcome. Gender was entered in Block 1, followed by cognitive reappraisal and expressive suppression in Block 2. The models were interpreted as conditional cross-sectional associations. The retained tables contain multiple *R*, model *F*, standardized beta, and *p* values; they do not contain the complete unstandardized coefficients, standard errors, confidence intervals, adjusted *R*^2^, *F*-change tests, or diagnostic statistics requested during review. These quantities cannot be reconstructed reliably from rounded summary values and have therefore not been fabricated.

Finally, an item-level latent-variable model was estimated in LISREL v8.80 using the 36 questionnaire items as observed indicators of the ERQ, SWLS, and DASS-21 constructs. The Satorra–Bentler statistic reported in the archived output was used for robust fit evaluation. Model fit was assessed using scaled chi-square, RMSEA with its 90% confidence interval, CFI, TLI/NNFI, NFI, RFI, GFI, AGFI, and SRMR (Kline, 2016). The model is highly complex relative to *N* = 106, so the estimates and fit indices are treated as exploratory. The archived output does not provide a reproducible table of standardized structural coefficients, standard errors, confidence intervals, or indirect effects. Consequently, the manuscript reports global fit only and does not claim that mediation was demonstrated.

Given the cross-sectional and non-experimental design, all findings were interpreted in terms of association rather than causation or temporal prediction. Table 5 summarizes the analytical strategy.

**Table 5:** Summary of the statistical analysis strategy.

Analytical phase	Statistical technique	Purpose
Descriptive analysis	Means and standard deviations	To describe the levels of life satisfaction, psychological distress, and emotion regulation strategies in the sample.
Bivariate analysis	Pearson correlations	To examine the direction, magnitude, and significance of associations among emotion regulation, depression, anxiety, stress, and life satisfaction.
Conditional association analysis	Hierarchical regression models	To assess whether cognitive reappraisal and expressive suppression explained additional outcome variance after adjustment for gender.
Structural analysis	Item-level latent-variable modeling	To evaluate global fit of the theoretical structure; indirect effects were not formally tested.
Model fit evaluation	Satorra–Bentler χ^2^, RMSEA, CFI, TLI/NNFI, NFI, RFI, GFI, AGFI, and SRMR	To assess the global fit and relevant limitations of the proposed structural model.

**Figure 2:**
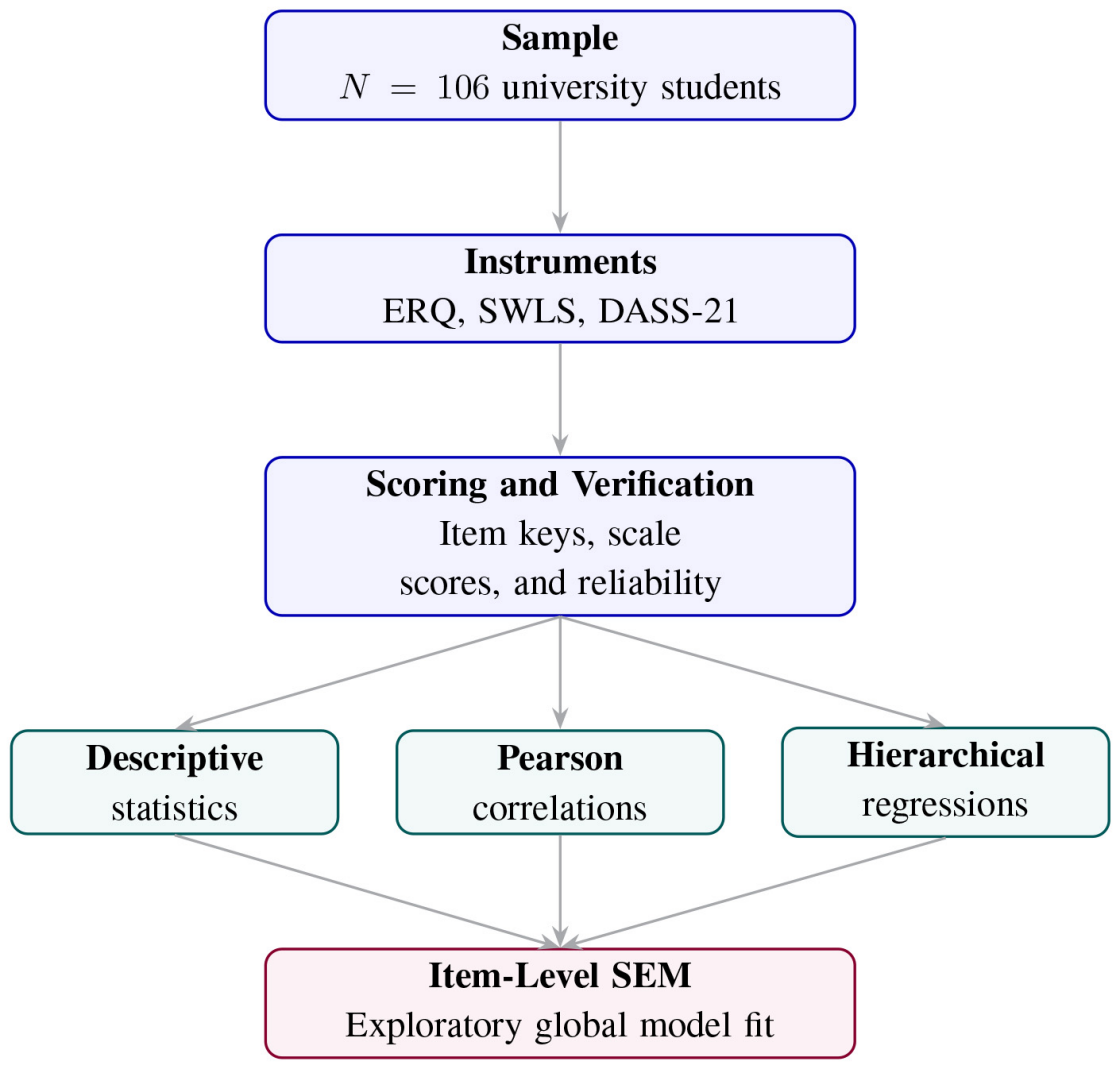
Methodological and analytical workflow of the study.

Figure 2 summarizes the methodological and analytical workflow.

## 3 Results

### 3.1 Descriptive analysis

Table 6 presents the descriptive statistics for the main study variables. The results provide an initial profile of the participants' subjective wellbeing, psychological distress, and emotion regulation strategies. Because the instruments used in the study have different response ranges, the descriptive values should be interpreted within the metric of each scale rather than as directly comparable scores across all variables.

**Table 6:** Descriptive statistics of the main study variables.

Variable	*M*	*SD*	Scale range	Instrument	Interpretative note
Life satisfaction	3.85	0.80	1–7	SWLS	Moderate global cognitive evaluation of life.
Depression	0.86	0.68	0–3	DASS-21	Lowest mean score among the three distress dimensions.
Anxiety	1.08	0.73	0–3	DASS-21	Intermediate level within the distress dimensions.
Stress	1.50	0.80	0–3	DASS-21	Highest mean score among the distress dimensions.
Cognitive reappraisal	3.82	0.83	1–7	ERQ	Moderate reported use of reinterpretation strategies.
Expressive suppression	4.17	1.04	1–7	ERQ	Slightly higher reported use of inhibition of emotional expression.

Scores should be interpreted according to the response range of each instrument. DASS-21 dimensions were assessed on a 0–3 scale, whereas SWLS and ERQ scores were assessed on a 1–7 scale.

Life satisfaction showed a moderate mean score (M = 3.85; SD = 0.80), suggesting an intermediate level of global cognitive evaluation of life among the participating students. This result indicates that, on average, students did not report extremely low life satisfaction, although the score does not reflect a highly elevated level of subjective wellbeing either. Therefore, the sample may be characterized by a moderate level of perceived life satisfaction within the academic context analyzed.

Regarding psychological distress, stress showed the highest mean score among the DASS-21 dimensions (M = 1.50; SD = 0.80), followed by anxiety (M = 1.08; SD = 0.73) and depression (M = .86; SD = .68). This pattern suggests that stress-related symptomatology was relatively more prominent than anxiety and depressive symptomatology in the sample. From a university wellbeing perspective, this finding is relevant because stress may represent an early indicator of emotional overload associated with academic demands, time pressure, assessment situations, and adaptation to higher education. Although the descriptive nature of these results does not allow causal interpretation, the higher relative presence of stress supports the need to examine how students regulate emotions in demanding academic contexts.

With regard to emotion regulation strategies, expressive suppression showed a higher mean score (M = 4.17; SD = 1.04) than cognitive reappraisal (M = 3.82; SD = 0.83). This result indicates that participants reported a slightly greater tendency to inhibit or modulate the external expression of emotions than to cognitively reinterpret emotionally relevant situations. However, this pattern should be interpreted cautiously. A higher score in expressive suppression does not necessarily imply maladaptive functioning in all cases, as the meaning and consequences of suppression may depend on the context, frequency of use, and flexibility with which the strategy is applied. Similarly, the level of cognitive reappraisal should be interpreted as an indicator of reported use rather than as direct evidence of regulatory effectiveness.

**Figure 3:**
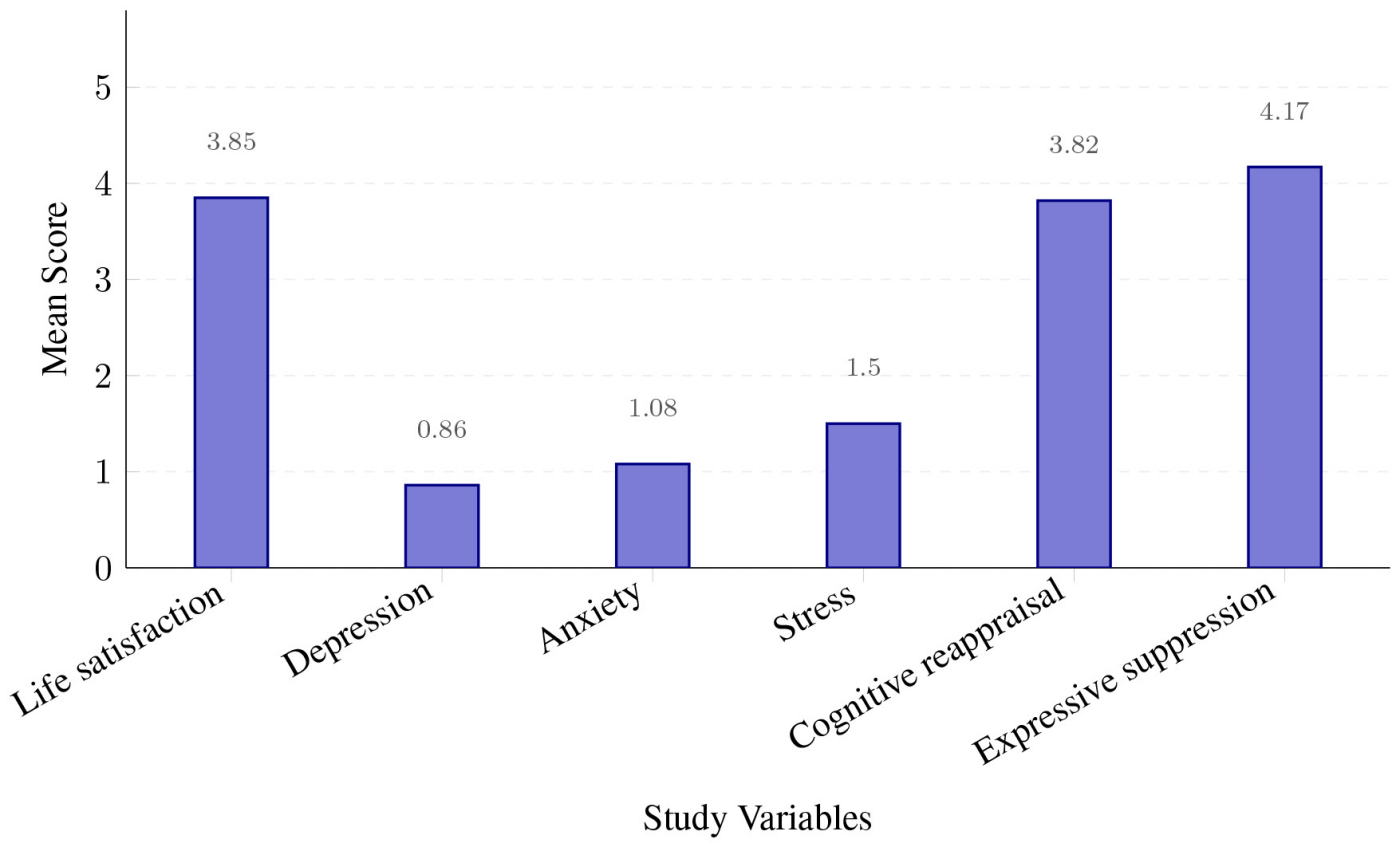
Descriptive profile of the main study variables. Bars represent means and vertical lines represent standard deviations. Scores should be interpreted within the response range of each instrument.

Overall, the descriptive results suggest a psychological profile characterized by moderate life satisfaction, a relatively higher presence of stress compared with anxiety and depression, and a slightly higher reported use of expressive suppression than cognitive reappraisal. Figure 3 visually summarizes this descriptive pattern.

### 3.2 Bivariate correlations

Pearson correlation analyses were conducted to examine the direction and magnitude of the associations among life satisfaction, psychological distress, and emotion regulation strategies. Table 7 presents the correlation matrix for the six main study variables.

**Table 7:** Pearson correlations among the study variables.

Variable	1	2	3	4	5	6
1. Life satisfaction	—					
2. Depression	−0.51^**^	—				
3. Anxiety	−0.34^**^	0.59^**^	—			
4. Stress	−0.40^**^	0.61^**^	0.79^**^	—		
5. Cognitive reappraisal	0.00	0.12	0.20^*^	−0.07	—	
6. Expressive suppression	0.06	0.05	−0.57^**^	−0.22^*^	0.59^**^	—

^*^p < 0.05, ^**^p < 0.01. Correlations should be interpreted as bivariate associations and not as causal relationships.

Life satisfaction showed negative and statistically significant associations with the three dimensions of psychological distress. Specifically, life satisfaction was negatively associated with depression (*r* = −0.51, *p* < 0.01), anxiety (*r* = −0.34, *p* < 0.01), and stress (*r* = −0.40, *p* < 0.01). These results indicate that students who reported higher levels of depressive symptomatology, anxiety, and stress tended to report lower life satisfaction. The strongest association was observed between life satisfaction and depression.

The three DASS-21 dimensions were positively and significantly interrelated. Depression was positively associated with anxiety (*r* = 0.59, *p* < 0.01) and stress (*r* = 0.61, *p* < 0.01), while anxiety showed a strong positive association with stress (*r* = 0.79, *p* < 0.01). This pattern suggests the presence of a shared psychological distress core in the sample, with anxiety and stress showing the highest degree of overlap.

With regard to emotion regulation strategies, cognitive reappraisal showed a positive and statistically significant association with anxiety (*r* = 0.20, *p* < 0.05), while its associations with life satisfaction, depression, and stress were not statistically significant. The positive association with anxiety should be interpreted cautiously, as it may reflect greater regulatory effort among students experiencing higher anxiety rather than a harmful effect of reappraisal.

Expressive suppression showed a negative and statistically significant association with anxiety (*r* = −0.57, *p* < 0.01) and stress (*r* = −0.22, *p* < 0.05), as well as a positive association with cognitive reappraisal (*r* = 0.59, *p* < 0.01). The negative association between expressive suppression and anxiety is especially notable because its direction and magnitude diverge from the pattern commonly reported in the literature. Although all subscales showed high internal consistency, reliability alone does not rule out scoring, labeling, or data-entry problems; the item coding and correlation matrix should therefore still be audited against the original statistical files.

**Figure 4:**
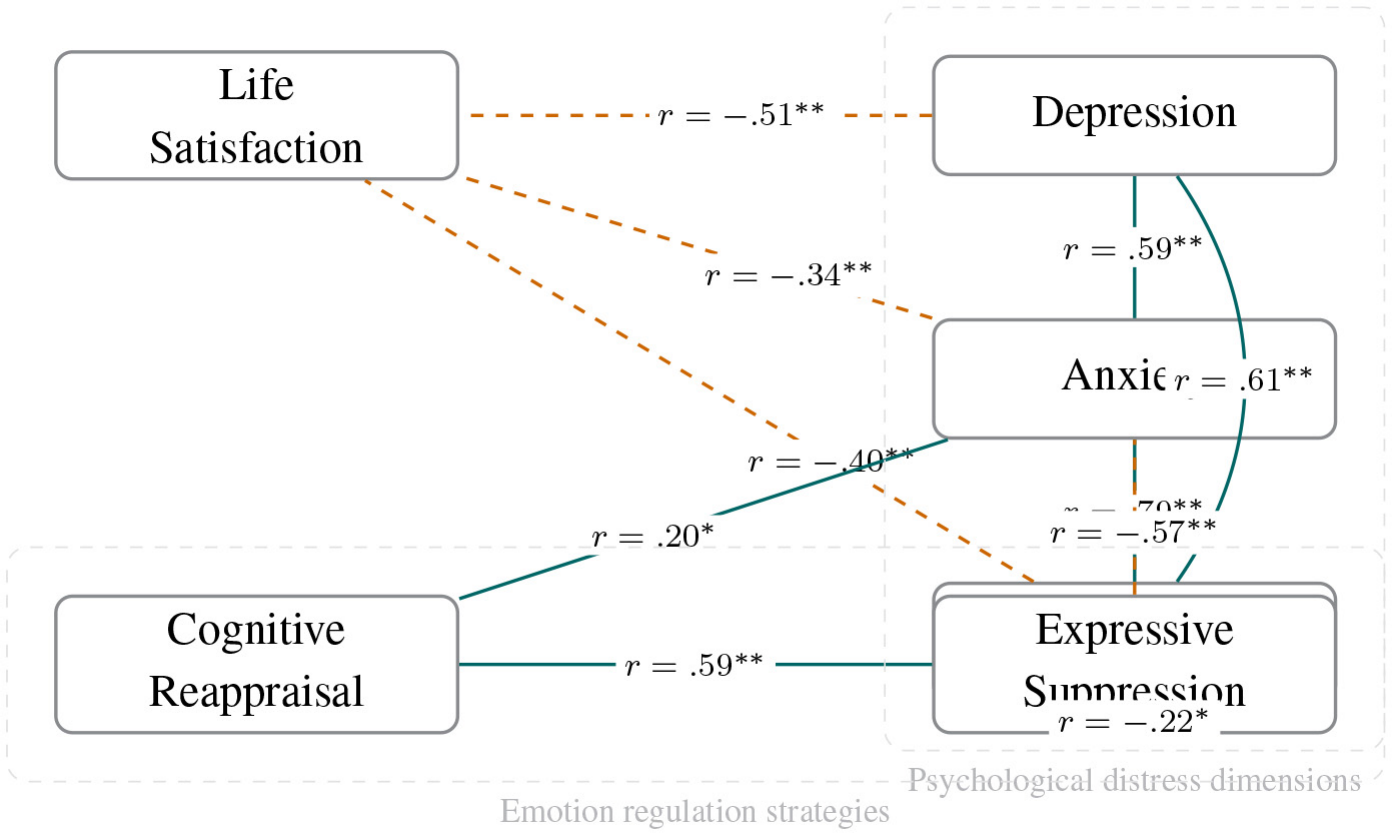
Network-style synthesis of statistically significant bivariate correlations. Continuous lines represent positive associations and dashed lines represent negative associations; no directionality is implied. **p < 0.01.

Overall, the bivariate results show three main patterns. First, psychological distress was consistently associated with lower life satisfaction. Second, depression, anxiety, and stress were strongly interrelated, supporting the presence of a common distress structure. Third, the associations between emotion regulation strategies and psychological adjustment were less linear than initially expected, particularly in the case of expressive suppression. Figure 4 provides a visual synthesis of the statistically significant bivariate associations.

### 3.3 Hierarchical regression analyses

**Figure 5:**
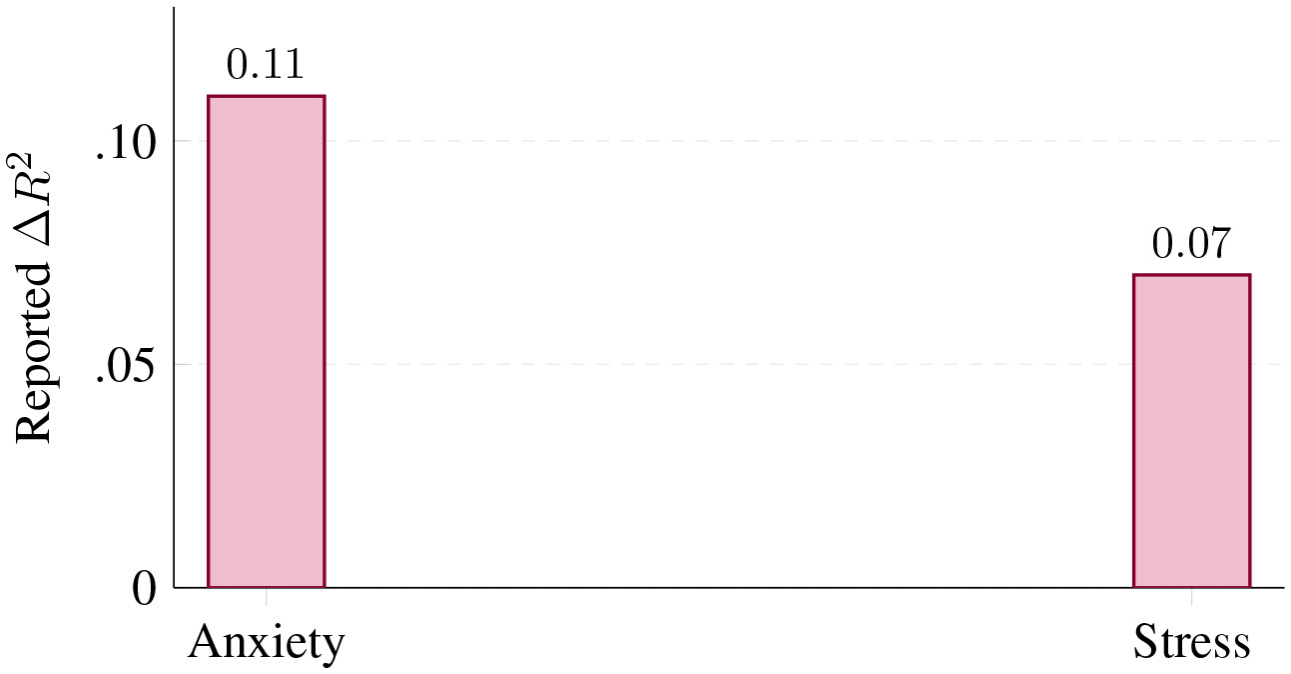
Reported increments in explained variance for the two statistically significant Block 2 regression comparisons. Exact *F*-change values were not available in the retained output. The figure shows the value of the increment *R*^2^ Δ*R*^2^

Hierarchical regression analyses examined whether emotion-regulation scores accounted for additional cross-sectional variance in psychological distress and life satisfaction after adjustment for gender (see Table 8). Four models were estimated, with depression, anxiety, stress, and life satisfaction as outcomes (see Table 9). Gender was entered in Block 1 and cognitive reappraisal and expressive suppression in Block 2 (see Table 10 and Figure 5).

**Table 8:** Hierarchical regression models for depression, anxiety, and stress.

Criterion	Block	Predictor	*R*	*F*	β	*p*
Depression	Block 1	Gender	0.06	0.46	−0.67	0.49
	Block 2	Cognitive reappraisal	0.14	0.75	0.15	0.22
		Expressive suppression	0.14	0.75	−0.03	0.75
Anxiety	Block 1	Gender	0.13	1.93	−0.13	0.16
	Block 2	Cognitive reappraisal	0.33	4.22	0.37	< 0.001
		Expressive suppression	0.33	4.22	−0.28	0.021
Stress	Block 1	Gender	0.12	1.54	−0.12	0.21
	Block 2	Cognitive reappraisal	0.27	2.74	0.09	0.41
		Expressive suppression	0.27	2.74	−0.29	0.01

*R* is the multiple correlation and β is standardized. Values are reproduced from the retained original table. The gender coefficient reported as β = −0.67 in the depression model and all other entries should be checked against the original SPSS output before submission.

**Table 9:** Reported incremental variance from the hierarchical regressions.

Outcome	*ΔR* ^2^	Reported significance	Interpretation
Depression	Not reported	n.s.	No significant increase was reported after adding the ERQ scores.
Anxiety	0.11	*p* < 0.01	The two ERQ scores accounted for additional cross-sectional variance.
Stress	0.07	*p* < 0.01	The two ERQ scores accounted for additional cross-sectional variance.
Life satisfaction	Not reported	n.s.	No significant increase was reported after adding the ERQ scores.

Exact adjusted *R*^2^, *F*-change statistics, and confidence intervals were not available in the retained output and have not been reconstructed from rounded values.

**Table 10:** Hierarchical regression model for life satisfaction.

Block	Predictor	*R*	*F*	β	*p*
Block 1	Gender	0.11	1.28	0.11	0.25
Block 2	Cognitive reappraisal	0.13	0.63	−0.05	0.67
Expressive suppression	0.13	0.63	0.09	0.43

The emotion-regulation variables were not significantly associated with life satisfaction in this model. Complete unstandardized coefficients, standard errors, confidence intervals, adjusted *R*^2^, and diagnostics were not available in the retained output.

**Figure 6:**
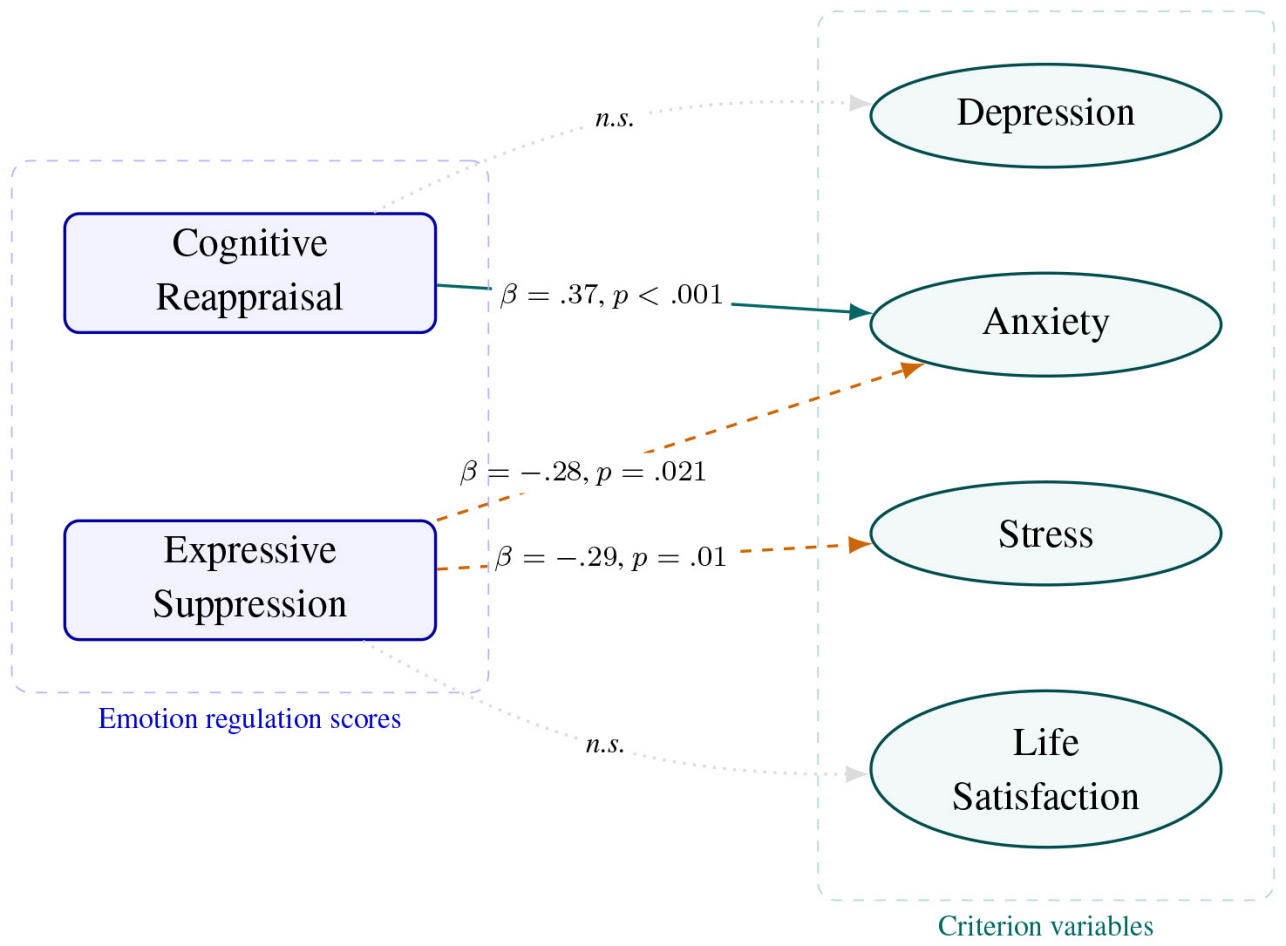
Synthesis of the hierarchical regression findings. Continuous arrows indicate positive adjusted associations, dashed arrows indicate negative adjusted associations, and dotted arrows indicate non-significant patterns.

In the depression model, neither block provided statistically significant evidence of association. Neither cognitive reappraisal nor expressive suppression was significantly associated with depressive symptomatology. In the anxiety model, gender was not statistically significant in Block 1. Adding cognitive reappraisal and expressive suppression accounted for an additional 11% of variance (Δ*R*^2^ = 0.11, *p* < 0.01). Cognitive reappraisal was positively associated with anxiety (β = 0.37, *p* < 0.001), whereas expressive suppression was negatively associated with anxiety (β = −0.28, *p* = 0.021). In the stress model, adding the two emotion-regulation scores accounted for an additional 7% of variance (Δ*R*^2^ = 0.07, *p* < 0.01). Expressive suppression was negatively associated with stress (β = −0.29, *p* = 0.01), whereas cognitive reappraisal was not significant (β = 0.09, *p* = 0.41). These unexpected directions require cautious interpretation and verification against the original scoring and analysis files (Figure 6).

Finally, neither gender nor the two emotion-regulation strategies was significantly associated with life satisfaction. Cognitive reappraisal (β = −0.05, *p* = 0.67) and expressive suppression (β = 0.09, *p* = 0.43) did not explain significant additional variance. Overall, the regressions provide partial support for H1, but the coefficients describe conditional associations rather than causal or temporal effects.

### 3.4 Structural equation model

A structural equation model was estimated to evaluate the integrated theoretical structure. The model used the 36 questionnaire items as observed indicators and included the ERQ, DASS-21, and SWLS constructs. This item-level specification is parameter intensive for *N* = 106 and is retained only as an exploratory analysis of global fit. It cannot establish temporal ordering or causality.

Fit evidence was mixed: $\chi_{SB}^{2}\left(506\right)=756.94$, *p* < 0.001; RMSEA = 0.068, 90% CI [0.058, 0.078]; CFI = 0.96; and TLI/NNFI = 0.95, whereas NFI = 0.88, RFI = 0.86, GFI = 0.69, AGFI = 0.61, and SRMR = 0.10. Thus, favorable incremental indices coexist with substantial absolute and residual misfit. The model should not be described as confirmed or as demonstrating an explanatory pathway. Table 11 summarizes the main fit indices obtained for the structural model.

**Table 11:** Fit indices for the structural equation model.

Fit index	Observed value	Interpretation
$\chi_{\text{SB}}^{2}$	756.94, *df* = 506, *p* < 0.001	Significant chi-square indicates imperfect exact fit, although this index is sensitive to model complexity and sample characteristics.
RMSEA	0.068, 90% CI [0.058, 0.078]	Reasonable approximation error; compatible with acceptable model fit.
CFI	0.96	Good incremental fit.
TLI/NNFI	0.95	Good incremental fit.
NFI	0.88	Slightly below the conventional acceptable threshold.
RFI	0.86	Below the conventional acceptable threshold.
GFI	0.69	Low absolute fit; suggests model limitations.
AGFI	0.61	Low adjusted fit; suggests model limitations.
SRMR	0.10	Above the recommended cut-off; indicates residual misfit.

Fit indices must be interpreted jointly. Low GFI and AGFI and an SRMR of 0.10 indicate relevant limitations despite favorable RMSEA, CFI, and TLI/NNFI. The sample is small relative to the item-level model complexity.

The global-fit results are consistent with some elements of the theoretical framework but do not establish individual structural relations. Standardized path coefficients, standard errors, confidence intervals, and indirect effects were not available in the archived output. In particular, model fit alone does not demonstrate mediation. No mediation conclusion is therefore drawn, and indirect effects should be tested with bootstrap confidence intervals in a future reproducible analysis.

**Figure 7:**
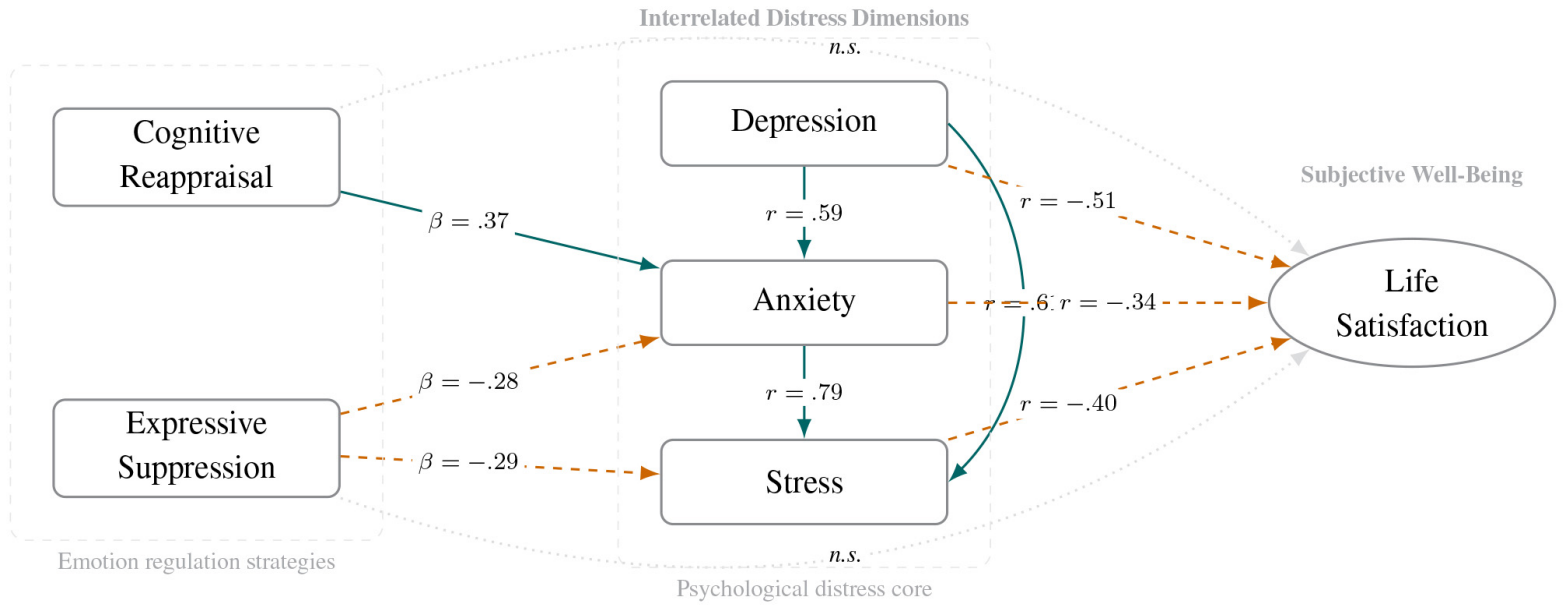
Visual synthesis of the main correlational and adjusted-regression findings. Continuous lines indicate positive associations, dashed lines indicate negative associations, and dotted lines indicate non-significant adjusted associations. Arrow direction reflects model organization and does not establish causality.

Figure 7 provides a visual synthesis of the main correlational and adjusted-regression findings.

## 4 Discussion

The aim of this study was to analyze associations between emotion regulation and university students' psychological wellbeing, considering depressive symptomatology, anxiety, stress, and life satisfaction simultaneously. Emotion-regulation scores were associated with some dimensions of psychological distress, particularly anxiety and stress, but not with depression or life satisfaction in the hierarchical regression models. The structural model yielded mixed global-fit evidence and is interpreted as exploratory.

The descriptive results showed that stress was the dimension of psychological distress with the highest relative presence in the sample, followed by anxiety and depression. This finding is consistent with previous studies identifying academic workload, examinations, time management, and uncertainty as prominent university stressors (Restrepo et al., 2020; Khorasani et al., 2023). It is also compatible with the multicentre findings of Micheluzzi et al. (2025), who found moderate-to-high perceived stress and prominent academic pressure in 2,103 Italian students. However, the present convenience sample is much smaller and more homogeneous, so it cannot support prevalence estimates or broad institutional generalization.

The author-supplied reliability estimates showed high internal consistency across all dimensions: alpha coefficients ranged from 0.846 to 0.921 and omega coefficients from 0.848 to 0.921. These results address the psychometric reporting request but do not, by themselves, resolve the unexpected direction of several ERQ associations.

Life satisfaction was negatively associated with depression, anxiety, and stress, supporting the fourth hypothesis of the study. This finding is consistent with the subjective wellbeing framework proposed by (Diener et al. 1985), according to which life satisfaction represents a global cognitive evaluation of one's own life that tends to deteriorate when negative emotional states increase. The strongest association was observed between life satisfaction and depression, suggesting that depressive symptoms may have a particularly relevant impact on students' global evaluation of their lives. This result is theoretically coherent, as depression usually involves discouragement, lack of interest, low positive affect, hopelessness, and negative self-evaluation, all of which may directly compromise the way students perceive their personal and academic experience.

The strong positive associations among depression, anxiety, and stress also deserve attention. These results suggest the presence of a shared core of psychological distress in the sample, especially between anxiety and stress. This pattern is consistent with transdiagnostic approaches that emphasize common mechanisms underlying emotional disorders, such as negative emotionality, affective reactivity, persistent worry, avoidance, and difficulty recovering emotional balance after demanding experiences (Bullis et al., 2019). In the university context, stress and anxiety may be particularly intertwined because academic demands often combine perceived overload, uncertainty, performance evaluation, and anticipatory concern. Therefore, interventions focused on university mental health should not address these dimensions as completely isolated problems, but as interrelated expressions of emotional maladjustment.

Regarding the first hypothesis, the results provide only partial support. Emotion regulation strategies explained a significant proportion of variance in anxiety and stress, but not in depression or life satisfaction. This finding suggests that the regulatory processes assessed through the ERQ may be more directly related to states of activation, tension, and worry than to broader depressive processes or global life evaluation. Depression may depend more strongly on other cognitive and emotional mechanisms not included in this study, such as rumination, helplessness, self-blame, reduced reinforcement, low perceived social support, or lack of academic meaning (Aldao et al., 2010; Lazarus and Folkman, 1984). Similarly, life satisfaction may be influenced by a broader set of variables, including perceived competence, social relationships, resilience, family support, economic conditions, institutional climate, and future expectations.

Cognitive reappraisal showed a positive relationship with anxiety, both in the bivariate correlation and in the regression model. This result diverges from the classical interpretation of cognitive reappraisal as an adaptive and protective strategy (Gross and John, 2003; Hu et al., 2014). However, this finding can be understood from a contextual and functional perspective. In a cross-sectional sample exposed to academic demands, higher scores in cognitive reappraisal may not necessarily indicate effective emotional regulation, but rather a greater effort to reinterpret situations already perceived as threatening or emotionally demanding. In other words, students with higher anxiety may engage more frequently in reappraisal attempts because they face more situations that require emotional management. Therefore, the positive association between cognitive reappraisal and anxiety should not be interpreted as evidence that reappraisal increases anxiety, but as an indication that the relationship between regulatory effort and emotional distress may be more complex than expected.

This interpretation is consistent with contemporary models of regulatory flexibility, which argue that the effectiveness of a strategy depends on the context, the timing of its implementation, the intensity of the emotional response, and the individual's ability to select strategies according to situational demands (Bonanno and Burton, 2013). Cognitive reappraisal may be protective when it is used flexibly and early in the emotion-generative process, but it may be less effective when the emotional response is already intense or when the situation is perceived as uncontrollable. In academic contexts, students may attempt to reframe examinations, workload, or interpersonal difficulties, but such attempts may coexist with high levels of anxiety if the underlying demands remain unresolved.

Expressive suppression also showed an unexpected pattern, as it was negatively associated with anxiety and stress. This finding partially diverges from previous literature, which usually describes expressive suppression as a less adaptive strategy when used frequently or chronically (Gross and John, 2003; Hu et al., 2014). Several explanations may be considered. In specific academic or social situations, suppression may function as a short-term strategy for maintaining composure, and some students may interpret it as control of external expression rather than emotional avoidance. Nevertheless, self-report bias, sample characteristics, scoring, and variable-label errors remain alternative explanations. The high reliability coefficients do not rule out these possibilities, so the original item coding and statistical output should still be audited. The third hypothesis, which proposed a negative association between emotion regulation and psychological distress, was therefore only partially supported. The findings do not allow a simple interpretation in which cognitive reappraisal is uniformly protective and expressive suppression is uniformly maladaptive. Instead, they point to a more nuanced pattern in which emotion regulation strategies may operate differently depending on the type of distress analyzed, the academic context, and the way students understand and report their emotional regulation processes. This is relevant because it suggests that research and intervention should move beyond a rigid classification of strategies and focus instead on regulatory flexibility, contextual adequacy, and the functional meaning of each strategy.

The second hypothesis was not supported: neither cognitive reappraisal nor expressive suppression was significantly associated with life satisfaction in the adjusted regression. Life satisfaction is likely related to a broader set of personal and contextual factors, including academic self-efficacy, social support, resilience, perceived control, motivation, family relationships, institutional support, and economic stability (Cohen and Wills, 1985; Connor and Davidson, 2003).

The structural model cannot be used to support mediation. Although RMSEA, CFI, and TLI/NNFI were favorable, GFI, AGFI, NFI, RFI, and SRMR indicated important limitations, and no indirect effects or bootstrap confidence intervals were reported. The SEM therefore provides only an exploratory assessment of global fit. The observed bivariate relationships between distress and life satisfaction should not be converted into an explanatory or temporal pathway.

Table 12 summarizes the degree of support for the study hypotheses.

**Table 12:** Summary of hypothesis support based on the main findings.

Hypothesis	Expected relationship	Support	Interpretation
H1	Emotion-regulation strategies are associated with anxiety, depression, stress, and life satisfaction.	Partially supported	Adjusted associations were observed for anxiety and stress, but not depression or life satisfaction.
H2	Greater cognitive reappraisal is associated with higher life satisfaction.	Not supported	Cognitive reappraisal and expressive suppression were not significantly associated with life satisfaction.
H3	Emotion regulation is negatively associated with depression, anxiety, and stress.	Partially supported	The observed pattern was mixed; cognitive reappraisal was positively associated with anxiety, while expressive suppression was negatively associated with anxiety and stress.
H4	Depression, anxiety, and stress are negatively associated with life satisfaction.	Supported	All three dimensions of psychological distress were significantly and negatively associated with life satisfaction.
Exploratory indirect association	Distress may be statistically intermediate between emotion regulation and life satisfaction.	Not tested	No indirect effects or bootstrap confidence intervals were available; mediation is not demonstrated.

Overall, the findings invite a more nuanced understanding of emotion regulation in university students. Cognitive reappraisal and expressive suppression should not be interpreted as universally adaptive or maladaptive strategies. Rather, their effectiveness appears to depend on regulatory flexibility, timing, emotional intensity, contextual demands, and the specific goal pursued by the student. This view is consistent with contemporary models of emotion regulation flexibility (Bonanno and Burton, 2013) and has important implications for psychological and educational interventions in higher education. From an applied perspective, the results suggest that university-based wellbeing programs should not be limited to promoting isolated emotion regulation techniques. Instead, they should help students develop broader emotional competencies, including the ability to identify emotional states, understand situational demands, choose appropriate regulatory strategies, seek social support, manage academic stress, and recognize when professional help is needed. Such programs should also avoid presenting suppression or reappraisal in overly simplistic terms. The key educational goal should be to promote flexible, context-sensitive, and reflective emotion regulation rather than the mechanical use of a single strategy. In summary, the present study contributes to the understanding of psychological wellbeing in university students by showing that emotion regulation is relevant but insufficient on its own to explain students' emotional adjustment and life satisfaction. Psychological distress, especially depression, anxiety, and stress, plays a central role in students' subjective wellbeing. The findings support the need for comprehensive explanatory models that integrate emotion regulation, distress processes, personal resources, and academic-contextual variables.

### 4.1 Educational and psychological implications

The findings of this study have several educational and psychological implications for the promotion of wellbeing in higher education. First, the results suggest that university wellbeing programs should not be limited to teaching isolated emotion regulation techniques. Although cognitive reappraisal and expressive suppression are relevant strategies for understanding emotional functioning, the results indicate that their relationship with psychological adjustment is complex and context-dependent. Therefore, interventions should move beyond the idea that one strategy is always adaptive and another is always maladaptive. Instead, universities should promote flexible, reflective, and context-sensitive emotion regulation.

From an educational perspective, emotion regulation should be understood as a transversal competence that supports academic adaptation, interpersonal functioning, and psychological wellbeing. Students are frequently required to manage emotions in situations involving examinations, oral presentations, academic overload, social comparison, uncertainty about the future, and interaction with teachers and peers. In this context, it may be useful to train students not only to reinterpret stressful situations, but also to identify emotional states, understand situational demands, evaluate available resources, select appropriate coping strategies, and seek support when necessary.

The results also highlight the relevance of stress as a central indicator of university maladjustment. Stress showed the highest descriptive score among the three dimensions of psychological distress, suggesting that it may function as an early warning signal. For this reason, universities should implement preventive actions aimed at identifying and reducing academic overload before it develops into more intense anxiety or depressive symptomatology. These actions may include psychoeducational workshops, mentoring programs, academic guidance, time-management training, and early screening of emotional distress.

From a psychological perspective, the findings support the need for comprehensive interventions that integrate emotional literacy, cognitive reappraisal training, coping skills, stress management, rumination prevention, social support, resilience, and academic self-efficacy. Such interventions should help students understand not only what they feel, but also why they feel it, what function the emotion may have, and which regulatory strategy is most appropriate for a given situation. This approach is consistent with the perspective of regulatory flexibility, according to which psychological adjustment depends not only on the use of specific strategies, but also on the ability to select, combine, and modify them according to contextual demands (Bonanno and Burton, 2013).

The unexpected associations observed in this study also have practical implications. Cognitive reappraisal was positively associated with anxiety, which suggests that students with higher anxiety may be making greater efforts to reinterpret demanding situations. Therefore, intervention programs should not assume that the mere use of reappraisal is sufficient. Students may need additional support to apply reappraisal effectively, especially when emotional activation is intense or when academic demands remain unresolved. Similarly, expressive suppression was negatively associated with anxiety and stress in this sample. Rather than interpreting this result as evidence that suppression is universally beneficial, it may indicate that some students use suppression as a short-term strategy to maintain composure in academic or social situations. Consequently, interventions should help students distinguish between occasional, functional emotional control and chronic emotional inhibition that may become harmful over time.

At the institutional level, the findings suggest that universities should develop multi-level wellbeing strategies. These strategies should combine individual-level interventions, such as emotional skills training and psychological counseling, with group-level and institutional actions, such as peer support, mentoring, teacher training, workload monitoring, and the design of emotionally supportive learning environments. Promoting student wellbeing should not be the responsibility of psychological services alone, but a shared institutional commitment involving teaching staff, academic advisors, student support units, and university governance.

Table 13 summarizes the main educational and psychological implications derived from the findings.

**Table 13:** Educational and psychological implications of the study findings.

Area of implication	Practical recommendation	Rationale based on findings
Emotion regulation training	Promote flexible use of different regulation strategies rather than teaching isolated techniques.	Cognitive reappraisal and expressive suppression showed a complex and non-linear pattern of association with distress.
Stress prevention	Implement early detection and prevention programs focused on academic stress.	Stress was the dimension of psychological distress with the highest descriptive score.
Psychological support	Integrate emotional literacy, coping skills, rumination prevention, and help-seeking behaviors into student support programs.	Emotion regulation alone did not fully explain depression or life satisfaction, indicating the need for broader interventions.
Academic guidance	Provide mentoring, time-management training, and support for academic planning.	Academic demands, overload, and uncertainty may contribute to emotional distress in university students.
Social and institutional support	Strengthen peer support, teacher-student communication, and institutional wellbeing policies.	Life satisfaction may depend on broader contextual resources beyond individual regulation strategies.
Intervention design	Avoid presenting cognitive reappraisal as always adaptive or expressive suppression as always maladaptive.	The observed results suggest that the function of each strategy depends on context, frequency, and flexibility of use.

In practical terms, the main implication of this study is that university interventions should aim to develop emotionally competent and flexible students rather than simply promoting the use of one preferred regulation strategy. Students should be supported in recognizing emotional demands, understanding their own regulatory patterns, and selecting strategies that are appropriate to the specific academic, social, or personal situation they are facing. This type of intervention may contribute to more realistic, inclusive, and effective wellbeing programs in higher education.

## 5 Limitations and future research

The cross-sectional design precludes causal and temporal inference. Expressions such as influence, effect, and mediation are therefore inappropriate for these data. Longitudinal or experimental studies are required to determine directionality and to test whether changes in emotion regulation precede changes in distress or life satisfaction.

The convenience sample was small (*N* = 106), drawn from one degree program, and predominantly female. These features constrain precision, representativeness, and generalizability and are particularly consequential for the item-level SEM. The mixed fit indices, including SRMR = 0.10, GFI = 0.69, and AGFI = 0.61, should be interpreted as relevant model limitations rather than overridden by favorable incremental indices.

All variables were self-reported. Although present-sample alpha and omega coefficients are now reported, the retained statistical archive did not contain complete regression statistics and diagnostics, documented missing-data decisions, exact gender contrast coding, standardized SEM coefficients, or indirect-effect tests. These omissions limit replicability. The unexpected directions of several ERQ associations must therefore remain provisional until the original item-level data and complete SPSS/LISREL outputs are independently audited.

Future research should use larger, more gender-balanced and multicentre samples; preregister scoring and missing-data rules; assess regression assumptions; and use parsimonious observed-composite or adequately powered latent models. Any indirect association should be tested with bootstrap confidence intervals and replicated longitudinally. Academic pressure, coping, social support, resilience, and regulatory flexibility should also be incorporated, consistent with recent multicentre evidence (Micheluzzi et al., 2025). Table 14 summarizes the main limitations of the study and the corresponding recommendations for future research.

**Table 14:** Main limitations and recommendations for future research.

Limitation	Impact on interpretation	Future research recommendation
Cross-sectional design	Prevents causal inference and temporal interpretation of the relationships among variables.	Conduct longitudinal studies to examine changes over time and clarify directionality.
Small and homogeneous sample	Limits precision and generalizability and is inadequate for stable estimation of a complex item-level SEM.	Replicate the study with larger and more diverse university samples and a more parsimonious model.
Unequal gender distribution	May influence the interpretation of emotional expression, distress, and regulation patterns.	Use more balanced samples and examine gender as a moderator.
Exclusive use of self-report measures	May introduce social desirability, response bias, and subjective interpretation of items.	Combine questionnaires with qualitative, behavioral, ecological, or physiological measures.
Unexpected direction of some findings	Requires caution in the interpretation of cognitive reappraisal and expressive suppression despite high internal consistency.	Audit scoring, labels, data entry, and analysis syntax and replicate the findings independently.
Limited set of regulation strategies	May reduce the explanatory capacity of the model, especially for depression and life satisfaction.	Include additional strategies such as rumination, acceptance, avoidance, catastrophizing, and problem solving.
Mixed SEM fit and absent indirect-effect tests	The proposed model is not confirmed and mediation is not demonstrated.	Test parsimonious alternative models and estimate indirect effects using bootstrap procedures.

## 6 Conclusion

Life satisfaction was negatively associated with depression, anxiety, and stress, while emotion-regulation associations were inconsistent with a simple adaptive-vs.-maladaptive distinction. Cognitive reappraisal was positively associated with anxiety, and expressive suppression was negatively associated with anxiety and stress. These sample-specific findings should not be interpreted causally or generalized beyond the study context.

The item-level SEM yielded mixed fit evidence and did not include formal indirect-effect tests. It therefore does not demonstrate mediation. The most defensible conclusion is that emotion regulation and psychological distress were associated with university wellbeing in this sample, but the direction and stability of the ERQ relationships require verification and replication.

Practical implications should remain cautious. Universities may consider broader, evidence-based support addressing academic pressure, coping, help-seeking, social support, and flexible emotion regulation, but the present study does not establish that any particular intervention will improve outcomes.

## Data Availability

The datasets presented in this article are not readily available because the dataset is not publicly available due to ethical and privacy restrictions related to participant confidentiality. Aggregated or anonymized data may be made available by the corresponding author upon reasonable request and subject to institutional and ethical approval. Requests to access the datasets should be directed to ahernand@ujaen.es.
